# Charcot–Marie–Tooth Disease with Myelin Protein Zero Mutation Presenting as Painful, Predominant Small-Fiber Neuropathy

**DOI:** 10.3390/ijms25031654

**Published:** 2024-01-29

**Authors:** Franco Gemignani, Antonio Percesepe, Francesca Gualandi, Isabella Allegri, Maria Federica Bellanova, Andi Nuredini, Elena Saccani, Enrico Ambrosini, Valeria Barili, Vera Uliana

**Affiliations:** 1 European Diagnostic Center, Polyclinic Dalla Rosa Prati, 43126 Parma, Italy; 2 Medical Genetics Unit, Department of Medicine and Surgery, University of Parma, 43126 Parma, Italy; 3 Medical Genetics Unit, University Hospital of Parma, 43126 Parma, Italy; 4 Medical Genetics Unit, Department of Mother and Child, Sant’Anna University Hospital of Ferrara, 44121 Ferrara, Italy; 5 Neurology Unit, Department of Specialized Medicine, University Hospital of Parma, 43126 Parma, Italy; 6 Laboratory of Neuromuscular Histopathology, Department of Medicine and Surgery, University of Parma, 43126 Parma, Italy; 7 Neurology Unit, Department of Medicine and Surgery, University of Parma, 43126 Parma, Italy

**Keywords:** Charcot–Marie–Tooth disease, myelin protein zero, neuropathic pain, skin biopsy

## Abstract

Charcot–Marie–Tooth disease (CMT) rarely presents with painful symptoms, which mainly occur in association with myelin protein zero (*MPZ*) gene mutations. We aimed to further characterize the features of painful neuropathic phenotypes in *MPZ*-related CMT. We report on a 58-year-old woman with a longstanding history of intermittent migrant pain and dysesthesias. Examination showed minimal clinical signs of neuropathy along with mild changes upon electroneurographic examination, consistent with an intermediate pattern, and small-fiber loss upon skin biopsy. Genetic testing identified the heterozygous variant p.Trp101Ter in *MPZ*. We identified another 20 CMT patients in the literature who presented with neuropathic pain as a main feature in association with *MPZ* mutations, mostly in the extracellular *MPZ* domain; the majority of these patients showed late onset (14/20), with motor-nerve-conduction velocities predominantly in the intermediate range (12/20). It is hypothesized that some *MPZ* mutations could manifest with, or predispose to, neuropathic pain. However, the mechanisms linking *MPZ* mutations and pain-generating nerve changes are unclear, as are the possible role of modifier factors. This peculiar CMT presentation may be diagnostically misleading, as it is suggestive of an acquired pain syndrome rather than of an inherited neuropathy.

## 1. Introduction

Charcot–Marie–Tooth disease (CMT) encompasses a genetically heterogeneous constellation of hereditary sensorimotor polyneuropathies, with a classical, distinctive phenotype of distal-muscle wasting and weakness with foot deformities [1,2]. Pain is probably not uncommon (including nociceptive pain related to osteoarticular disorders) [3,4]; however, CMT patients with predominating painful manifestations have rarely been reported and mainly occur in association with mutations in the myelin protein zero (*MPZ*) gene [5], which are classified as CMT1B-CMT2I/J subtypes.

Here, we report on a CMT patient with a p.Trp101Ter *MPZ* mutation presenting with neuropathic pain likely related to small-fiber damage demonstrated by skin biopsy.

## 2. Materials and Methods

### 2.1. Case Study

Besides a neurological examination, the clinical assessment included the CMT Neuropathy Score version 2 (CMTNS2) [6]. CMTNS2 is a nine-item scale based on the assessment of sensory and motor symptoms and signs in the legs and arms (CMT Examination Score—CMTES; maximum 28 points for 7 items), the ulnar/median compound motor-action potential (CMAP), and the radial sensory-action potential (SNAP) (electrophysiology score; maximum 8 points for 2 items).

Two neuroalgologic questionnaires, Douleur Neuropathique en 4 questions (DN4) [7] and the Neuropathic Pain Symptom Inventory (NPSI) [8,9], and a screening tool for small-fiber neuropathy (SFN), the Small-Fiber Neuropathy Symptoms Inventory Questionnaire (SFN-SIQ) [10], were also administered.

An electroneurographic (ENG) study with standard methods was performed using the Nicolet EDX System with Viking Software version 22.0.2.146 (Viking EDX, Natus, WI, USA). Motor-nerve-conduction studies were performed with surface electrodes. The CMAP latency, amplitude, area, duration, and motor-conduction velocities were recorded. Minimal, mean, and maximal F responses, latency, and persistence were obtained. Sensory-nerve-conduction studies for upper limbs were performed with the orthodromic method, stimulating at the fingers with ring electrodes and recording with surface electrodes at the wrist. For lower limbs, sural-nerve antidromic-conduction studies were performed with surface electrodes. The SNAP peak latency, amplitude, duration, and conduction velocity were calculated. All findings were compared with normal values from our laboratory.

Three mm punch skin biopsies were performed at the distal leg and proximal thigh after the appropriate consent was obtained. Biopsy specimens were processed and immunostained using the PGP9.5 antibody for intraepidermal nerve-fiber density (IENFD) evaluation according to published guidelines [11].

Written informed consent for genetic testing was obtained from the patient. Next-generation sequencing (NGS) analysis was performed using a custom gene panel that included 80 genes for inherited neuropathies (NEUROMIO panel—the list of genes is available upon request) by Illumina technology (Nextera Rapid capture-Illumina, San Diego, CA, USA) on a MiSeq-dx sequencer (Illumina, San Diego, CA, USA). Data were analyzed and filtered using Variant Interpreter version 2.16.0.235 (https://variantinterpreter.informatics.illumina.com (accessed on 11 November 2021), Illumina, San Diego, CA, USA). The Exome Variant Server (ESP; https://bio.tools/exome_variant_server, accessed on 11 November 2021), the Exome Aggregation Consortium (ExAC; http://exac.broadinstitute.org/, accessed on 11 November 2021) and the gnomAD database (https://gnomad.broadinstitute.org/, accessed on 11 November 2021) with a frequency greater than 0.1% were used to filter out common variants. Molecular confirmation of the identified variant was performed by standard Sanger sequencing on an automated analyzer (ABI PRISM 3500DX, ThermoFisher, Waltham, MA USA).

### 2.2. Review of the Literature

Reports about patients with *MPZ* mutations and pain were collected from a search on PubMed, using the search terms Charcot–Marie–Tooth disease/*MPZ* mutations/CMT1B/CMT2I/CMT2L, and pain. Relevant information included clinical data, with a focus on the features of pain and motor impairment, ENG findings, and molecular CMT diagnosis. When adequate clinical data were available, pain was classified as neuropathic based on the plausible neuroanatomical distribution and suggestive reported features (“descriptors”). Furthermore, we evaluated the clinical reports in order to state whether the phenotype was predominantly characterized by neuropathic pain or by motor symptoms and signs (including sensory ataxia). ENG patterns were classified based on the ulnar-nerve motor-nerve-conduction velocities (MNCVs) (or other upper limb MNCVs), as either demyelinating (substantially slowed MNCV) or axonal (normal, or near-normal MNCVs); an MNCV between 35 and 45 m/s was defined as intermediate [12].

## 3. Results

### 3.1. Case Report

A 58-year-old woman had a longstanding history of intermittent migrant pain and dysesthesias, mainly in the feet and legs but also in the thighs and arms. Mild to moderate symptoms of tenderness or myalgias were chronically present, but exacerbations characterized by burning pain and shock-like sensations, lasting some days or weeks, periodically occurred, with the pain intensity peaking at 8 in the numerical rating scale (NRS). One such exacerbation episode, at age 57, occurred after a second dose of the mRNA-based SARS-CoV2 vaccine (Pfizer-BioNTech BNT162b2, Pfizer, New York, NY, USA). Nocturnal cramps and/or the urge to move the legs when in bed were cyclically present, with features consistent with restless legs syndrome (RLS). 

The definite onset of symptoms occurred at age 46, with an initial diagnosis of fibromyalgia; however, nocturnal myalgias in the calves were occasionally reported in the previous years, as well as “rheumatic pains” during infancy. The subject past medical history was otherwise unremarkable; in particular, there were no neuropathy risk factors such as diabetes, obesity, malnutrition, alcohol abuse, or neurotoxic medications.

Family history was also unremarkable, except for a deceased paternal uncle reportedly affected with polyneuropathy. The proband’s mother had mild cognitive impairment but no neuromuscular symptoms and signs, and the ENG was normal. A 61-year-old brother was affected with Parkinson’s disease, while her 26-year-old son was asymptomatic. At the time of genetic counselling, there were no close family members available for genetic testing; thus, no segregation study was performed.

On examination, there was no muscle wasting or weakness, but some hypertrophy of the calves and slight pes cavus were seen and deep tendon reflexes in the lower limbs were evoked after facilitation maneuvers.

The subject scored 4/10 in the DN4 questionnaire, which was consistent with neuropathic pain. The NPSI questionnaire revealed a predominance of paroxysmal (7/10) and superficial burning (5/10) qualities of pain, with a total NPSI score of 24/100. The SFN-SIQ was 26/39, more than the proposed cut-off value of 6.5 [13], and the CMTNS2 was 4/36, consistent with a mild form of CMT. Laboratory tests for the following were normal or negative: the comprehensive metabolic profile; complete blood count, thyroid-stimulating hormone, hemoglobin A1C, vitamin B12 and folate levels, homocysteine, serum protein electrophoresis with immunofixation, rheumatoid factor, antinuclear antibody, double-stranded DNA, HCV antibody, Lyme antibody, and C-reactive protein.

The ENG showed reduced MNCVs of 38 m/s (left) and 37 m/s (right) in the peroneal nerves (normal > 45 m/s), 34 m/s (left) and 36 m/s (right) in the tibial nerves (normal > 45 m/s), 35 m/s in the left median nerve (normal > 50 m/s), and 48 m/s in the left ulnar nerve (normal > 50 m/s), with normal compound motor-action potential (CMAP) amplitudes. Sensory nerve-conduction velocities (SNCV) were reduced in the sural nerves (28 m/s left; 25 m/s right; normal > 38 m/s), in the left median nerve (27 m/s; normal > 40 m/s), and the left ulnar nerve (26 m/s; normal > 40 m/s), with normal sensory-nerve action potentials (SNAPs). The ENG pattern was classified as intermediate, although the ulnar MNCV was near-normal, as the other MNCVs were in the intermediate range.

**Figure 1:**
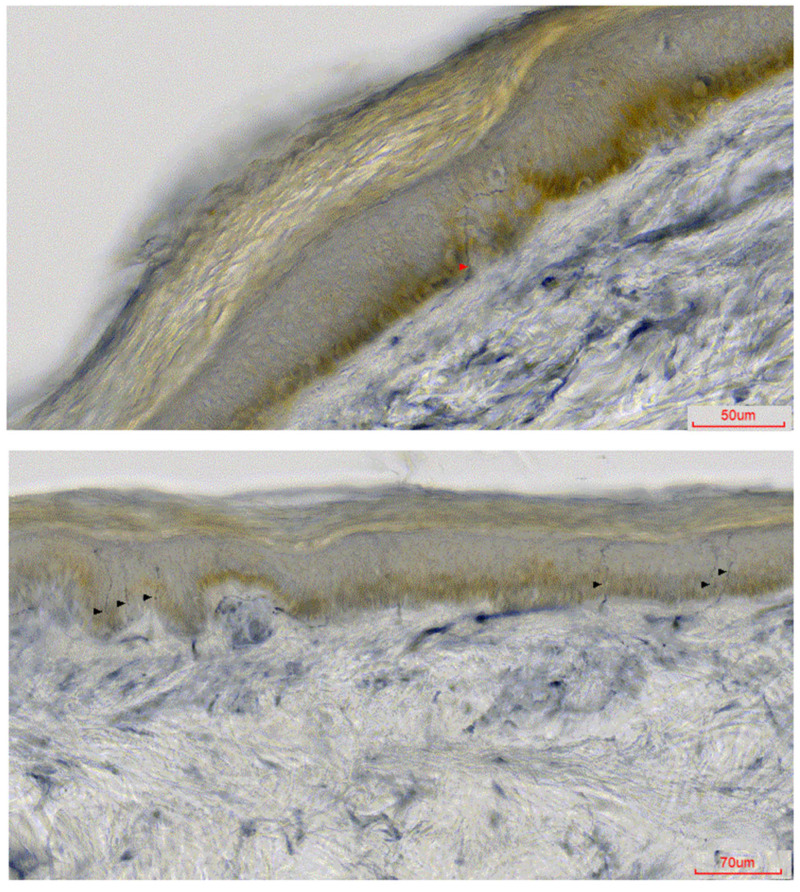
Bright-field immunohistochemistry of 50 μm thick sections of the skin biopsy immunostained with protein gene product 9.5 (PGP 9.5). There are long epidermal areas devoid of intraepidermal nerve fibers at the thigh (**upper**) and at the ankle (**lower**). The few remaining PGP 9.5-positive nerve fibers crossing the dermal–epidermal junction are indicated by arrowheads.

The skin biopsy, performed at age 56, revealed a reduced IENF density (Figure 1) at the ankle (3.5 IENF/mm; normal > 4.03 [11]) and at the thigh (2.2 IENF/mm; normal value of our laboratory > 9.6). The findings were deemed to be consistent with non-length-dependent SFN; the case was classified as predominant SFN, considering that mild clinical and ENG signs of large-fiber involvement were additionally present.

The MLPA (multiplex ligation-dependent probe amplification) method for *PMP22* analysis indicated a normal copy number for the gene, excluding CMT1A. The NGS analysis using the NEUROMIO panel identified the heterozygous variant NM_000530.8:c.302G>A (p.Trp101Ter) in *MPZ*. The variant was rated as pathogenic according to the American College of Medical Genetics and Genomics (ACMG) criteria [14] by attributing PVS1 (a null variant in a gene whose loss of function is a known mechanism of disease), PP5 (a variant recently reported by a reputable source as pathogenic), and PM2 (absent from control). In the ClinVar database, this variant is classified as pathogenic (one star), and it has been cited in the literature as being associated with CMT [5].

### 3.2. Cases in the Literature

**Figure 2:**
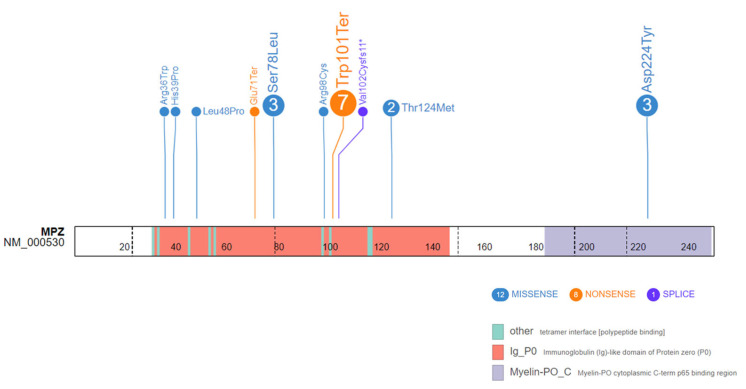
MPZ protein representation displaying variants associated with painful neuropathy. The numbers within the circles correspond to probands mentioned in the 20 literature-based cases plus the present case report, which share identical constitutional variants. Circle colors are indicative of the variant type (missense, nonsense, and splicing), while protein-domain regions are depicted using distinct colors.

We found 23 articles reporting on 42 CMT patients with *MPZ* mutations and pain symptoms. In 20 patients (13 women, 6 men, 1 non-specified) from 11 families [5,15,16,17,18,19,20,21,22,23,24], painful symptoms of neuropathic pain predominated (Table 1), with absent or mild motor signs; severe motor impairment or sensory ataxia was the main clinical feature in the remaining cases, which were not included. The majority of patients had adult onset (range 8–47 years; median 30 years), although onset was within the second decade in six patients. The ENG findings were classified as intermediate in 12 patients (including the present case), demyelinating in 5, and axonal in 2. The localization of variants in the *MPZ* gene is illustrated in Figure 2. Different mutations were found in each family, with the exception of the p.Trp101Ter mutation that was found in our patient and in the Ramirez family [5] and the p.Thr124Met mutation found in another two families [15,17].

## 4. Discussion

CMT associated with *MPZ* mutations is phenotypically and genotypically heterogeneous, as the *MPZ* protein is involved in various aspects of Schwann-cell biology [25,26]. Studies of phenotype–genotype correlations in *MPZ* variants showed that patients and mutations were separated into three subtypes: infantile onset with extremely slow MNCVs and severe motor impairment; gradual onset within the first two decades of life and slow MNCVs in the 20–25 m/s range; and adult onset, with normal or near normal MNCVs [27,28]. The phenotypes are almost completely mutation-specific, and *MPZ* mutations act in different ways to cause infantile, childhood, or adult-onset neuropathy, respectively resulting in the developmental disruption of myelination processes in early phases, the formation of mature myelin sheaths with abnormal structure and function, or defective Schwann-cell–axon signaling leading to axonal damage with preserved myelin sheaths [27,29].

The adult-onset phenotype has been characterized as classical CMT with axonopathic ENG features (CMT2I/J) [27,29]. However, the broader use of genetic testing in recent years, leading to the identification of an increasing number of *MPZ* mutations causing a late-onset phenotype, has revealed the occurrence of atypical presentations, such as neuropathy with liability to pressure palsies [30,31], sensory ataxia [32], predominance of positive sensory symptoms [33], and cranial nerve involvement [34], often with MNCVs in the intermediate range [28].

The painful phenotype of *MPZ* mutations is seemingly rare; however, this point has not been specifically addressed in large studies investigating genotype–phenotype correlations [27,29,34,35], and only a few cases from the literature are mentioned by Callegari et al. [28] in the setting of atypical MPZ phenotypes. In the literature, we found 20 cases from 11 families that harbored a total of 10 different mutations, with features similar to those of our patient (Table 1). Our patient’s mutation has been previously described in an English relation who also presented with neuropathic pain [5]. The truncating p.Trp101Ter variant is predicted to result in a loss of function, primarily attributed to its association with nonsense-mediated decay (NMD). This prediction is based on the variant’s characteristics; specifically, being a premature termination codon (PTC) located within a small exon that leads to the degradation of the transcript, preventing the synthesis of the corresponding protein. According to Lindeboom et al. (2019) [36], such a configuration, involving a PTC in a small exon (within exon 3 of 216 bp) situated more than 55 nucleotides away from a splicing site, is supportive of nonsense-mediated decay.

Recently, it was reported that nonsense-mediated decay mutations caused milder forms of CMT1B, and the underlying mechanism was haplo-insufficiency of MPZ and loss of function [32].

Considering that more than 200 *MPZ* mutations have been identified [34], it could be that nerve-fiber changes generating pain symptoms are associated only with definite variants.

In the majority of these patients, there was adult onset, with moderately slowed or near-normal MNCVs and minor CMT clinical signs, such as pes cavus and mild distal weakness. However, most reported patients with a painful phenotype harbored an *MPZ* mutation in the extracellular domain, which, in general, is more frequently involved. The only exception was a family with a missense mutation, c.700G>T p.Asp234Tyr [22]; the proband had clinical features consistent with a superimposed chronic inflammatory demyelinating polyneuropathy (CIDP).

Additional information can be inferred from the few neuropathological studies available. In our patient, the skin biopsy demonstrated small-fiber damage that likely underpinned the pain symptoms. This is a novel finding, as the IENF density was normal in another patient with the same mutation [5]. In this patient, doubtful aspects of dermal nerve-fiber demyelination were described as possibly related to the pain symptoms. In other patients, sural-nerve biopsy showed clusters of regenerating fibers, possibly acting as irritable nociceptors [17,22]. These findings, however, are not specific for *MPZ* mutations, nor obligatorily associated with pain symptoms [37].

On the other hand, it has been observed that phenotypic variations occur in the same *MPZ* mutation [21,38], even with intrafamilial clinical heterogeneity [38,39]. Interestingly, in the family described by Kilfoyle et al. [20], only the index case had neuropathic pain, whereas several other family members had RLS, a putative equivalent of neuropathic pain [40]. In our patient, manifestations of neuropathic pain and RLS coexisted, as well as in two patients reported by Schneider-Gold et al. [22]. 

The clinical expression of *MPZ* mutations might be influenced by modifier genes and acquired or environmental factors [29]. In particular, it has been suggested that the manifestations and the course of the disease in some patients are modified by a superimposed autoimmune/inflammatory response, possibly driven by the changed immunogenicity of an abnormal MPZ protein, in keeping with the experimental observations of secondary autoimmunity and CIDP-like manifestations in P0 knockout mice [41,42]. In some patients, consistent features of an acute/subacute onset [20], rapidly progressive [43] or stepwise-fluctuating course [19,44,45], elevation of cerebrospinal fluid protein [19,20,22,44,45,46], and response to immunotherapies [22,44,45,46] were described, including cases with painful neuropathy. Other comorbidities potentially influencing the neuropathy expression, such as diabetes or B12 deficiency that are often associated with painful neuropathy, are not mentioned in the case studies. A comprehensive view might be that the potential painfulness of nerve-fiber changes related to specific *MPZ* mutations can be unmasked or amplified by concomitant autoimmunity and/or inflammation. In our case, there was no evidence of superimposed acquired conditions, but the exacerbation of symptoms after vaccination might reflect a susceptibility to inflammatory/immune factors. The SFN following SARS-CoV2 vaccination [47,48] and an immuno-mediated disease flare [49] have been previously reported. In our patient, the painful symptoms and biopsy findings of small-fiber changes antedated vaccination, and we cannot exclude that recurrence of symptoms after vaccination was coincidental. 

The features of this CMT presentation may be misleading, as the clinical picture is reminiscent of other painful conditions, such as SFN and fibromyalgia, and only minor signs are seen upon neurological examination and ENG study. In the absence of obvious motor signs and of a suggestive family history, the differential diagnosis of SFN may be difficult on clinical grounds. Abnormalities in nerve-conduction studies, though mild, should not be overlooked as a possible clue to the CMT diagnosis.

In summary, the painful phenotype associated with *MPZ* mutations, though rare, is noteworthy for its possible diagnostic relevance in the context of neuropathic pain syndromes. As for the pathophysiology, it is unclear whether neuropathic pain is driven by definite nerve-fiber changes specifically related to definite *MPZ* mutations. Further studies of phenotype–genotype correlations should involve a larger number of neuropathic-pain patients harboring *MPZ* mutations, combining molecular studies with a comprehensive assessment of neuropathic pain. Patients with these features are probably under-recognized, and it is expected that they will be more frequently diagnosed in the future given the rise in genetic testing that is extended to patients with non-classical CMT features and a late onset. Along these lines, a painful CMT subtype associated with *MPZ* mutations was able to be more specifically characterized, which also contributes to the knowledge of neuropathic pain mechanisms in general, with a unique genetic model of peripheral neuropathic pain.

## Figures and Tables

**Table 1:** Patients with painful neuropathy and *MPZ* mutations.

Ref	Patient	Age at Onset	Pain Features	Distribution	Motor Signs	Associated Features	NCV	Nerve/Skin * Biopsy	Treatment	Family History	*MPZ* Mutation
[15]	PN-54 III.1 M	4th dec	Pains and paraesthesias	Lower limbs	Slight weakness of peroneal muscles	No	Axon	ND	NR	AD variable severity, hearing loss	Thr124Met c.371C>T
[16]	W 36 y	36 y	Burning—recurrent	Soles to lower legs	No weakness	No	Interm	De/remyelination, axonal loss, uncompacted myelin lamellae	NR	AD Father asymptomatic	Glu71ter c.211G>T
[17]	Index M 48 y	39 y	Dysesthesia	Feet	Normal	Unreactive pupils	Axon	Marked fiber loss, clusters	NR	AD Dysesthesia in 3, motor impairment variable	Thr124Met c.371C>T
[18]	Pt2 W 26 y	18 y	Pain	Feet and legs	No weakness	No	Interm	ND	NR	AD marked phenotypic variations in 5 members	Leu48Pro c.143T>C
[19]	M 47 y	47 y	Acute lancinating pain, recurrent	Arms and dorsal forearms, thighs and legs, to buttocks and face.	Normal strength	No	Interm	ND	IVIG ineffective	AD Mother paucisymptomatic	Arg36Trp c.106A>T
[20]	IV-103 W 30 y	30 y	Acute onset, severe burning and shock-like pain	More in the distal feet than in the hands	No	No	Interm	Normal myelinated fibre density	Pain management NS	AD- RLS in 8 of 10, hearing loss 7; asymptomatic 4	His39Pro c.116A>C
[21]	II-1 M 50 y	28 y	Severe cramps and painful paresthesia	NR	Mild weakness	No	Dem	ND	NR	AD family 2, all patients with cramps and painful paresthesia	Ser78Leu c.233C>T
	II-4 M 46 y	26 y	Severe cramps and painful paresthesia	NR	Moderate weakness	No	Dem	ND	NR		Ser78Leu c.233C>T
	III-3 M 11 y	8 y	Severe cramps and painful paresthesia	NR	Mild weakness	No	Dem	ND	NR		Ser78Leu c.233C>T
[22]	III-1 W 62 y	45 y	Painful sensations	Legs	Mild distal weakness	RLS	Interm	Mild large fiber loss, regeneration clusters	IVIg, gabapentin, pregabalin-improvement	AD RLS without pain in 1	Asp224Tyr c.670G>T
	IV-1 W 41 y	30 y	Cramps and pain	Legs	Mild distal weakness	No	Interm	ND	NR		Asp195Tyr c.670G>T
	IV-2 W 35 y	35 y	Cramps and burning pain	Legs and arms	Mild distal weakness	RLS	Interm	ND	NR		Asp195Tyr c.670G>T
[5]	Pt 1 W 18 y	1st dec	Deep, burning, aching, shooting, throbbing pain NRS 7–10	Feet, ankles	No	No	ND	ND	tricyclic antidepressants, gabapentinoids, opiates, lidocaine plaster—partially effective	AD Similar features in all	Trp101ter
	Pt 2 W 23 y	1st dec			Mild distal weakness	No	Interm	ND			Trp101ter c.302G>A
	Pt 3 W 25 y	2nd dec			No	No	Interm	ND			Trp101ter
	Pt 4 W 40 y	3rd dec			No	No	ND	ND			Trp101ter
	Pt 5 W 42y	3rd dec			No	No	Interm	Normal *			Trp101ter
	Pt 6 W 44 y	4th dec			No	No	Interm	ND			Trp101ter
[23]	F1-III.1 W 53 y	15 y	Burning	Lower extremities	Normal strength	No	Dem	ND	NR	AD Mild symptoms and signs in other 2	Val10Cysfs11 * c.309G >T
[24]	NA	Adult	Pain	Distal	No motor symptoms	No	Dem	ND	NR	NR	Arg98Cys c.292C>T
CR	W 57 y	46 y	Burning pain, shock-like sensations, NRS 8.	Mainly distal, occasionally proximal	No motor symptoms and signs	RLS	Interm	Abnormal *	Paracetamol, clonazepam—partial improvement	Sporadic	Trp101ter c.302G>A

Abbreviations: Ref—reference; CR—present case report; M—man; W—woman; dec—decade; y—years; Pt—patient; NR—not reported; ND—not done; NCV—nerve-conduction velocity; Axon—axonal; Interm—intermediate; Dem—demyelinating; NRS—numerical rating scale; AD—autosomal dominant; NA not available; *—skin biopsy; RLS—restless legs syndrome; IVIG—intravenous immunoglobulins.

## Data Availability

All relevant data are available from the corresponding author upon request.
